# Cognitive and Neurochemical Changes Following Polyphenol-Enriched Diet in Rats

**DOI:** 10.3390/nu13010059

**Published:** 2020-12-27

**Authors:** Margarita R. Ramis, Fiorella Sarubbo, David Moranta, Silvia Tejada, Jerònia Lladó, Antoni Miralles, Susana Esteban

**Affiliations:** 1 Laboratory of Neurophysiology, Biology Department, University of Balearic Islands (UIB), Ctra. Valldemossa Km 7, 5 E, 07122 Palma de Mallorca, Spain; margaramis87@gmail.com (M.R.R.); mariafiorella.sarubbo@hsll.es (F.S.); david.moranta@uib.es (D.M.); silvia.tejada@uib.es (S.T.); amiralles@uib.es (A.M.); 2 Research Unit, Hospital Universitario Son Llàtzer, Health Research Institute of the Balearic Islands (IdISBa), Carretera Manacor Km 4, 07198 Palma, Spain; 3 CIBERON (Physiopathology of Obesity and Nutrition), 28029 Madrid, Spain; 4 Health Research Institute of the Balearic Islands (IdISBa), 07120 Palma, Spain; 5 Department of Biology and University Institute of Health Sciences Research (IUNICS-IdISBa), University of Balearic Islands, 07122 Palma, Spain; jeronia.llado@uib.es

**Keywords:** memory, monoamines, SIRT1, dietary polyphenols, brain health, aging, antioxidant, 5-HT, NA, DA

## Abstract

Dietary recommendations are frequently developed based on nutrient deficiency or prevention of disease, but less attention has been paid to the dietary guidelines to promote brain health. Active and healthy aging is a prerequisite for improving quality of life as people age, and evidence is establishing a relationship between diet and brain health. This work studied the effect of a diet based on foods rich in antioxidants, especially polyphenols, in rats, three days a week for 20 months starting at 14 months. Behavioral analysis testing working memory, spatial and episodic memory, as well as brain monoaminergic neurotransmitters involved in these processes but also in general brain health were analyzed. In addition, hippocampal SIRT1 protein which has an important role in regulating normal brain function was evaluated. The results show that long-term intake of polyphenol-enriched diet improves memory and learning, correlating with restoration of brain monoaminergic neurotransmitters and hippocampal SIRT1 levels in aged rats. These results agree with reports revealing a neuroprotective effect of different polyphenolic compounds on age-related brain decline, based on its antioxidant and anti-inflammatory properties; and demonstrate that consumption of antioxidant-rich foods, a few days a week, gives good long-term results in terms of brain health.

## 1. Introduction

Aging is a normal physiological process caused by a set of mechanisms [1], mainly influenced by oxidative stress [2], systemic inflammation and also neuroinflammation [3], which influence each other, affecting the general physiology and the correct functionality of the brain. Over the past few decades, as life expectancy has increased significantly, cognitive decline has emerged as one of the greatest threats to health in old age [4] even in the absence of neurodegenerative diseases [4], which may compromise the ability of the elderly to maintain an active and independent lifestyle during normal aging [5]. For all these reasons, several studies suggest that healthy eating could protect the brain from these processes involved in aging [6] and nutritional psychiatry experts propose the growing role of diet in mental health [7].

It is plausible that dietary interventions can be used to improve brain health and for the treatment and prevention of brain aging and dementia [6]. An easy strategy to promote healthy brain aging is to increase the intake of some healthy foods, previously demonstrated to exert multiple favorable effects toward human health. For many years, plant-derived products have been used not only for nutritional benefits but also for potential properties that improve health and attenuate the development and progression of diseases. Several studies have shown that fruits, vegetables, and spices contain anti-oxidant and anti-inflammatory activities that can promote healthy aging [8], as well as decrease risk of chronic diseases [9] that are often associated with cognitive function. Since the end of the last century polyphenols have been proposed as possible molecules that can strategically prevent or slow down aging in general, and particularly brain aging. Polyphenols are compounds present in fruits and vegetable such as grapes, red fruits, or citrus fruits among others [10], whose antioxidant [11,12] and anti-inflammatory properties [13,14] have been widely described. Polyphenols have the ability to cross the blood brain barrier, due to their lipophilic nature [15] so they can protect the brain from oxidative stress and neuroinflammation [16,17,18,19,20]. They also induce activation of anti-aging enzymes like sirtuin 1 [21,22,23,24] which together seem to constitute the key points of their anti-aging effects. In fact, recent evidence from observational studies demonstrated that higher intake of dietary polyphenols is associated with better cognitive function in adults [25].

In relation to the above, it is now well known that old age in both animals and humans is associated with changes in brain regions such as the hippocampus [26]. Therefore, the present work aims to evaluate the effect of a diet enriched in polyphenols and other antioxidants on cognitive functions by using hippocampus-dependent behavioral tests, in rats as well as some neurochemical determinations in hippocampus and other brain regions. In this way, it aims to analyze at the same time the modulation of synthesis and metabolism of monoamines (5-HT, NA, and DA) and SIRT1 protein, which have an important role in regulating normal brain function. A diet rich in antioxidants could protect the oxidation of proteins such as sirtuins and limiting enzymes in the synthesis of monoamines with a consequent protection of cognitive functions and brain plasticity.

## 2. Materials and Methods 

### 2.1. Animals and Treatments

Eleven male Wistar rats (14 months; 535 ± 25 g, Charles River, Spain) were used. Animals were housed individually in standard cages and maintained under a 12 h light:12 h dark cycle (lights on at 08.00 h) and controlled environmental conditions (20 ± 2 °C; 70% humidity) with free access to tap water. One group of rats (*n* = 6) received an antioxidant enriched diet during 20 weeks, while the control group (*n* = 8) received standard food (Panlab A04, Spain) *ad libitum*. The same rats were studied throughout the study. The antioxidants enriched diet consisted of 30 g pellets (Panlab A04)/rat/day plus 20–25 g of food rich in antioxidants administered to each rat 3 times a week. These foods consisted of fruits and assorted vegetables, such as grapes, apples, plums, cherries, tomatoes, oranges, broccoli, carrots, walnuts, almonds; cereals such as oat, rice and wheat; juices of different red fruits and pineapple; green tea and red tea, among others (for more details, see Table S1 in supplementary material). The experimental diet has been designed on the basis of references indicating that, after dietary supplementation, concentrations of polyphenols or their metabolites reaching the brain are high enough to exert a pharmacological effect and physiological activity ([19], and other references therein). No changes between groups were observed in relation to the weight of the animals (data not shown; see supplementary material Figure S1; control animals = 569 ± 16 g, *n* = 8; diet treated animals = 588 ± 22 g, *n* = 6, at the end of the study).

Along the chronic treatment, cognitive abilities were checked (see next sections). Decapitation was performed to sacrifice the rats at 20 months during dark/light change (8:00 AM) when the behavior analysis was finished. So as to analyze the in vivo activity of limiting enzymes in monoamine synthesis, a single administration of 100 mg/kg, i.p. of NSD 1015 (central aromatic amino acid decarboxylase inhibitor,) was administered to all rats 30 min before the sacrifice, and the activity of the tyrosine hydroxylase (TH) and the tryptophan hydroxylase (TPH) were measured by means of the accumulation of dihydroxyphenylalanine (DOPA) and 5-hydroxytryptophan (5-HTP), respectively. Brains were quickly removed and dissected on an ice-cold plate in order to isolate the striatum (caudate-putamen) and the hippocampus from both cerebral hemispheres, and the pineal gland; and immediately, the tissues were frozen in liquid nitrogen and stored until analysis at −80 °C.

All procedures were performed in accordance with the European Convention for the Protection of Vertebrate Animals used for Experimental and other Scientific Purposes (Directive 86/609/EEC) and approved by the Bioethical Committee of the University (approval file number 2014/05/AEXP).

### 2.2. Behavioral Tests

Different tests depending on the function of the hippocampus and striatum were used. From 9:00 to 13:00 h the behavioral tests were performed. The rats were analyzed individually, and in order to avoid possible chronobiological masked effects, animals from the different experimental groups were alternated. Rats were located in the experimental room half an hour before tests for familiarization. To monitor cognitive and motor abilities evolution, several tests were conducted just previously to the treatment beginning, monthly, and at the end of treatment. In each session, the first test performed was the radial maze followed by the novel object recognition the second day, and the rotarod test the third day. The Barnes maze was only evaluated at the end of the treatment after carrying out the above-mentioned tests. To eliminate olfactory traces, the different devices were cleaned with ethanol before each test. 

#### 2.2.1. Spatial Working Memory in Radial Maze Test

As previously reported, spatial working memory in rats was evaluated by the 8-arm radial maze (Panlab) [27]. It consisted of an octagonal central platform (30 cm diameter) with eight radial arms (50 cm long, 10 cm wide) equally distributed and with visual clues in every arm entrance (stickers of diverse shapes and colors). The experimental room also had some visual cues on the walls, and small pieces of food pellets were put at the end of each arm. To achieve a convenient motivational level, rats were previously submitted to 48 h fasting [28]. So as to assess working memory, rats had to enter each arm to obtain the food and the test finished when all of them were visited or 20 min had elapsed in the trial. Errors consisted in the sum of non-visited arms and re-entry into arms. Movements of the animals and the distance traveled were monitored by a digital video tracking system (LE 8300 with software SEDACOM v 1.3, Panlab) which were analyzed with the software SMART v 2.5 (Panlab). 

#### 2.2.2. Visuospatial Learning in Barnes Holeboard Maze 

This test was carried out at the end of the treatment with the diet rich in polyphenols. The maze was elevated above the floor (75 cm) and consists of a 130 cm circular disk with 18 holes located around the perimeter equidistantly. A black box or target (30 × 20 × 10 cm) was located under one of the holes being not distinguishable from the other holes from the center of the maze. Several visual cues were included in the experimental room as reference points for locating the target. As a stimulus to find the target, a bright light was turned on to accentuate the natural agoraphobia of rats [29]. The rats were allowed to explore the maze freely the day before the test (familiarization phase). Rats were individually placed in the middle of the platform, the light was turned on and 3 min were given to the animals to escape by hiding in the target. If the animals did not escape, they were placed in the target box manually, where it stayed during 1 min. The light was turned off when the animal was inside the box. The day of the test, four trials separated by 10 min were performed by the rats. The trial was considered done when the animal reached the target, or after 3 min when animals were placed into the target box manually and remained there for 1 min. Latency was defined as the time used to explore the maze until reaching the target, and an error was considered and counted as the exploration of the non-target holes. Three different strategies to search the target were considered: direct (animals enter directly the target), serial (animals explore holes sequentially), or random (other patterns to reach the target) as described by Rueda-Orozco and cols. [30]. 

#### 2.2.3. Non Spatial Memory in Novel Object Recognition Test

New object recognition is a test to quantity episodic-like memory in rodents; it assesses the natural tendency of the rats to explore based on the spontaneous exploratory activity novelty [31]. An open field device (78 cm diameter cylinder with 60 cm high opaque wall) was used for the three phases performed: habituation, familiarization, and test phase [24,32]. The habituation phase was performed daily 4 consecutive days, and each rat explored the open field freely during 10 min without objects. The fifth day, each rat was located in the device for re-habituation by allowing it to explore for 1 min. Next, during the familiarization phase, animals were placed in the center of the apparatus with two identical objects (e.g., plastic blue cubes 6 × 6 cm) always placed in the same location (35 cm between them, 16 cm away from the walls). Until 10 min were reached, animals were allowed to inspect both objects. Object exploration implied the contact between the object and the nose or mouth of the animal. The test phase was carried out 10 min later, during which the animals were repositioned in the open field with one familiar object (one used in the previous phase previously cleaned) and one new object (e.g., a plastic red bolus 8 × 4 cm). During a 10 min period, the time used exploring the objects was recorded. The objects were made with the same material although with dissimilar shapes and they had never been related to reinforcement or location avoiding natural significance for the animals. In addition, it was verified in every training session that the time spent with both objects were similar as they were novel for the rats. Thus, the test phase reflects the preference for novelty. 

Ethanol solution was used to clean the objects and the device between trials. Furthermore, the number of times that the rats passed through the different areas of the open field and the number of urinations and defecations were recorded so as to measure anxiety and exploratory drive of the rats.

#### 2.2.4. Rotarod Test of Motor Coordination

The rotarod treadmill device (Panlab) allows to evaluate the motor ability and balance of the animals. Previous to the test, a training session/day was performed during 4 days on a rotarod at a 4 rpm constant speed until the rats were stabilized. In the test phase, an acceleration mode (from 4 to 40 rpm over a period of 60 s) was used so the rats were placed on the rotarod for recording the latency to fall. Every animal did five trials separated by a few minutes for the rat recovery, and competency was defined as the average time recorded. 

### 2.3. Neurochemical Analysis

In the current work, the long-term effects of the antioxidant-enriched diet on the functional synthesis and metabolism of the monoamines 5-HT, DA and NA were analyzed in brain regions involved in memory processes regulation and motor activity of aged rats that completed the behavioral trials. It was also evaluated the levels of the SIRT1 protein in hippocampus homogenates.

#### 2.3.1. Tryptophan Hydroxylase (TPH) and Tyrosine Hydroxylase (TH) Activity

In the current work, to evaluate the in vivo synthesis of 5-HT, DA, and NA in this study, the activity of tryptophan hydroxylase (TPH, tryptophan-5-monoxygenase, EC 1.14.16.4)—as the rate-limiting enzyme for 5-HT synthesis—and tyrosine hydroxylase (TH, tyrosine-3-monoxygenase, EC 1.14.16.2)—as the rate-limiting enzyme for DA and NA synthesis—were measured by determining the 5-HTP and DOPA accumulation within 30 min after inhibition of the aromatic L-amino acid decarboxylase (EC 4.1.1.28) by a maximally effective dose of NSD-1015 (100 mg/kg, i.p.) [27]. The 5-HTP and DOPA accumulation method is the most commonly used assay system to monitor the in vivo rate of tryptophan and tyrosine hydroxylation in the brain. Moreover, the pool of 5-HT, DA, and NA unaffected by new synthesis primarily stored neurotransmitter intraneuronally, and the metabolite levels let to analyze the recent use of these neurotransmitters. TPH exist in two isoforms: TPH-1 is mainly expressed in the pineal gland and in gut enterochromaffin cells [33] and TPH-2 is preferentially expressed in the brain, where it plays a fundamental role in 5-HT synthesis [34]. High-performance liquid chromatography with electrochemical detection (HPLC; Waters, Barcelona, Spain) was used to determine the 5-HTP and DOPA formed from endogenous tryptophan and tyrosine, respectively, and the other compounds in the hippocampus (5-HT and NA terminal-rich region) and corpus (dorsal) striatum (rich in 5-HT and DA nerve terminals). Consequently, these data related to the 5-HTP synthesis in hippocampus and striatum regions refer to the activity of the TPH-2 isoform, while the 5-HTP formed in the pineal gland shows 5-HT synthesis mediated by TPH-1. A common step in the synthesis of catecholamines is tyrosine hydroxylation, so the DOPA accumulation in the striatum preferentially indicates DA synthesis and the one in the hippocampus was mainly related to the NA synthesis. The output electric current was monitored by an interphase (Waters busSAT/IN Module) connected to a computer. The concentrations of the compounds in a given sample were calculated by interpolating the corresponding peak height into a parallel standard curve using the software Empower Pro (Waters). For more details of the procedure see Ramis and cols. [24]. 

#### 2.3.2. SIRT1 Protein by Western Blot Analysis

A 1:15 weight/volume of cold homogenization buffer (50 mM Tris-HCl, pH 7.5; 1 mM EDTA; 2% SDS) was used to homogenize the right hippocampus from the rats in the presence of a protease inhibitor cocktail (Pierce) with an Ultra-Turrax homogenizer (Type Tp 18/10, Janke and Kunkel, Germany) two times for 10 s each sample. Extracts were also sonicated two times for 10 s. Total protein content from homogenates were analyzed using the acid bicinchoninic method following manufacturer’s instructions (Pierce^TM^ BCA Proetin Assay Kit) and total protein content was adjusted to 6 µg/µL in each sample. Then homogenates were mixed 1:1 with loading Laëmmli buffer. Protein samples (40 μg) were separated by 10% SDS-PAGE and transferred to nitrocellulose membranes (3 MM Whatman). Immunoblot analyses were performed by using the antibodies: anti-SIRT1 (rabbit polyclonal, 1:1000 dilution, Merck-Millipore; Cat. #07-131) and anti-β-actin (mouse monoclonal, 1:10,000 dilutions, Sigma-Aldrich; Cat. A1978). Secondary HRP-linked antibodies consist of anti-rabbit IgG (Goat polyclonal, 1:5000 dilutions, Cell Signaling; Cat. #7074) and anti-mouse IgG (Horse polyclonal, 1:5000 dilutions, Cell Signaling; Cat. #7074). Proteins were detected using the ECL Western Blotting Detection Reagents (Amersham). The chemiluminescence bands were detected by exposure to photographic films (Hyperfilm Amersham) and digitalized with a GS-800 scanner, and the integrated optic density was analyzed with the QuantityOne software (Bio-Rad, Hercules, CA, USA). Every sample was analyzed no less than 3 times in different gels and membranes were reproved for β-actin; which was used for protein normalization. Unless otherwise stated, the other reagents were purchased from Merck group (Darmstadt, Germany).

### 2.4. Statistics

Graph-Pad Prism (version 6.0) was used for analysis of the data. Two-way or three-way ANOVA were used for statistical evaluation of behavioral results using Bonferroni post-hoc test for pairwise statistical comparisons; two-way repeated measures ANOVA was used when the same animals were compared throughout the treatment. One-way ANOVA and Student t-test were used for neurochemical evaluations. Results are expressed as mean ± S.E.M. and *p* ≤ 0.05 was considered statistically significant. 

## 3. Results

### 3.1. Cognitive and Motor Abilities of Rats along the Chronic Treatment 

**Figure 1:**
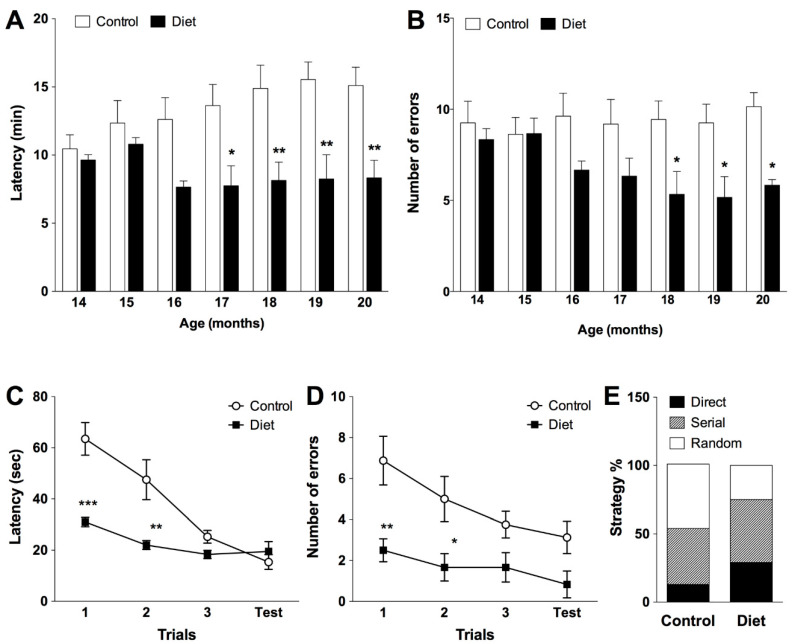
**Evolution of spatial working memory task along the chronic treatment assessed by eight-arm radial maze test** (**A**,**B**) in animals fed with the experimental diet (*n* = 6) and control animals (*n* = 8). Bars represent the mean ± SEM of latency (time needed to complete the test in minutes) (**A**), errors committed during the test (number) (**B**). (**A**) Two-way repeated measures ANOVA detected a significant effect for the treatment (F(1, 72) = 9.158, *p* = 0.0105), the subjects (F(12, 72) = 12.20, *p* < 0.0001) and the interaction (F(6, 72) = 4.434, *p* = 0.0007) but not for age (F(6, 72) = 1.70, *p* = 0.1334). (**B**) Two-way repeated measures ANOVA detected a significant effect for the treatment (F(1, 72) = 12.85, *p* = 0.0038) and subjects (F(12, 72) = 2.238, *p* = 0.0183), but not for age (F(6, 72) = 0.7766, *p* = 0.5909) or the interaction (F(6, 72) = 1.570, *p* = 0.1683); * *p* < 0.05, ** *p* < 0.01, *** *p* < 0.001 when compared treated and control group, by two-way repeated measures ANOVA followed by Bonferroni post-hoc test. **Assessment of spatial learning/memory in the Barnes maze test in old rats (20 months)** (**C**–**E**). Mean ± SEM derived from total latency (time spend to locate the target in seconds) (**C**) and number of errors committed during the test (**D**); and strategy followed to locate the target (**E**). (**C**) Two-way ANOVA detected a significant effect for the treatment F(1, 48) = 21.12, *p* < 0.0001; trial F(3, 48) = 16.61, *p* < 0.0001and interaction of both: F(3, 48) = 6.458, *p* = 0.0009. (**D**) Two-way ANOVA detected a significant effect for the treatment F(1, 48) = 23.44, *p* < 0.0001; trial F(3, 48) = 3.391, *p* = 0.0253 but not for interaction of both: F(3, 48) = 0. 7157, *p* = 0.5476. * *p* < 0.05, ** *p* < 0.01, *** *p* < 0.001 when compared treated group with control group, by two-way ANOVA followed by Bonferroni post-hoc test.

A clear improvement in spatial memory as a result of administration of the studied diet was observed. The polyphenol-enriched diet induced an improvement in the performance of the radial maze test; from the middle of the treatment, significant differences were detected among the two groups (Figure 1). At the end of the treatment, the diet group significantly reduced the time needed to complete the test (45%, Figure 1A) and committed fewer errors (40%, Figure 1B) compared to the control group.

Spatial learning was evaluated at the end of the treatment on old animals with the Barnes maze test. All animals learned to locate the target throughout the training sessions but the animals that received the experimental diet performed better than controls (Figure 1). In the first trials, animals fed the polyphenol-rich diet remembered the target location learned the previous day (familiarization phase) better than control animals, with a significant effect observed on latency by locating the target more quickly (Figure 1C) and making fewer errors (Figure 1D) than controls. This improvement in spatial learning in polyphenol-fed rats may be due to the predominant use of the direct and serial strategy while half of the control group followed a random strategy (Figure 1E).

**Figure 2:**
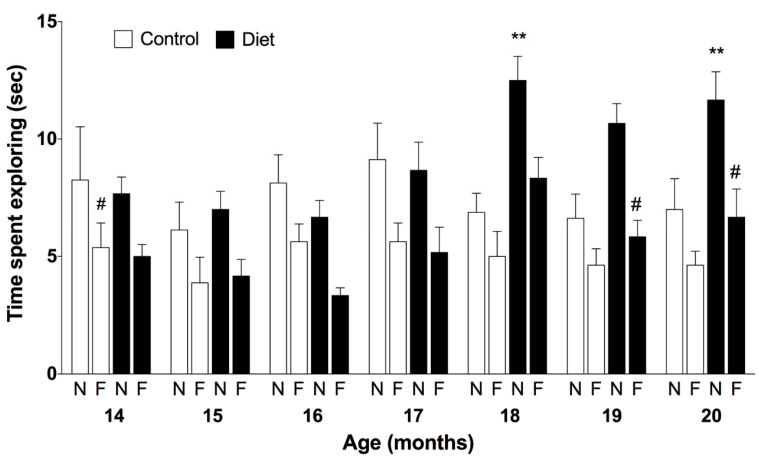
**Episodic memory along the chronic treatment assessed by the novel object recognition test.** Bars represent the mean ± SEM of time spend (in seconds) exploring the novel and the familiar objects during the test in seconds in animals fed with the experimental diet (*n* = 6) and control group (*n* = 8). Three-way ANOVA was followed by Bonferroni post-hoc test for the statistical analysis. Three-way ANOVA detected a significant effect for the treatment F(1168) = 7.87, *p* = 0.0056; age F(6168) = 3.027, *p* = 0.0078 and objects F(1168) = 55.51, *p* < 0.0001; ** *p* < 0.01 when compared novel object in the treated group vs. novel object in the control group at the same age point by Bonferroni post-hoc test; ^#^ *p* < 0.05 when compared novel vs. familiar object in the treated group at 19 and 20 months by Bonferroni post-hoc test.

A positive effect of the polyphenols-enriched diet was also observed in the open field device. An increase in exploration time was observed in animals receiving the polyphenol-rich diet, especially of the novel object compared to the control group (67%, Figure 2). However, the discrimination of objects did not reach the level of statistical significance.

No significant changes in motor coordination were observed between the animals chronically fed with the experimental diet and the controls (data not shown, see Supplementary Material Figure S2).

### 3.2. Effect of Polyphenol-Enriched Diet on Monoamine Synthesis and Metabolism in Hippocampus, Striatum and Pineal Gland

**Figure 3:**
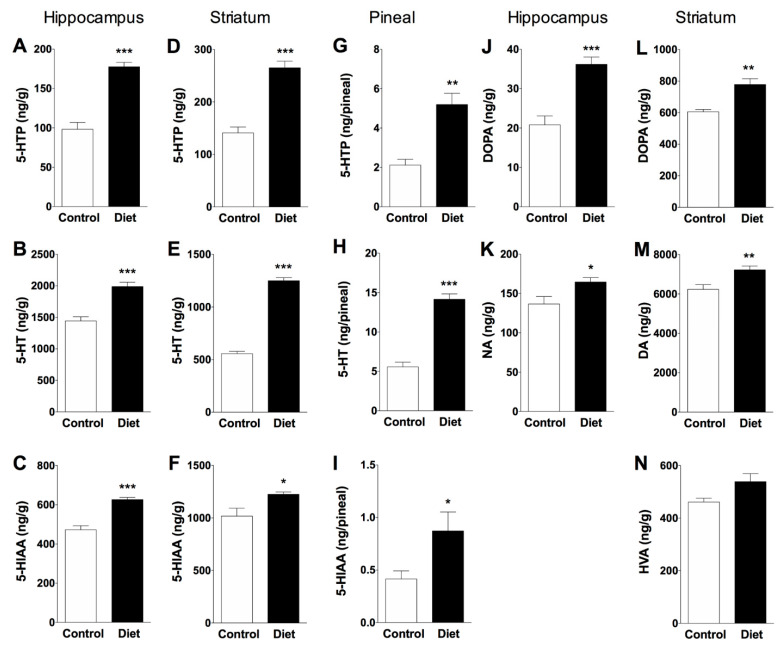
Effects of an antioxidant-enriched diet in serotonergic and catecholaminergic systems in hippocampus, striatum and pineal gland of old rats (20 months). Serotonergic system (**A**–**I** graphs): Bars represent the mean ± SEM (ng/g of wet tissue) in hippocampus and striatum or ng/pineal in overall pineal gland) of 5-HTP accumulated during 30 min after decarboxylase inhibition, 5-HT tissue content, and 5-HIAA metabolite values. Catecholaminergic systems (**J**–**N** graphs): Bars represent the mean ± SEM (ng/g of wet tissue) of DOPA accumulated during 30 min after decarboxylase inhibition, NA or DA tissue content in hippocampus and striatum, respectively, and HVA metabolite values in striatum. Unpaired t-test was used to statistical evaluation; * *p* < 0.05, ** *p* < 0.01, *** *p* < 0.001 when compared treated rats (*n* = 6) and control group (*n* = 5).

A positive effect was observed in the animals that received the diet rich in polyphenols in the three monoaminergic systems analyzed (5-HT, NA and DA) at the end of the treatment in the hippocampus and striatum, two important brain regions in different cognitive functions (Figure 3). A variation in tyrosine hydroxylation with greater accumulation of 5-HTP and DOPA over control group was clearly observed as a result of the administration of the diet rich in polyphenols, indicating increased activities of limiting enzymes THP-2, TPH-1 and TH respectively. The treatment with the experimental diet increased TPH-2 activity (measured as 5-HTP accumulation) in hippocampus (81%, Figure 3A) and striatum (88%, Figure 3D) over controls fed the standard diet. Consequently, the mean value of 5-HT content followed a similar pattern, it was increased after experimental diet in hippocampus and striatum (38% and 125%, Figure 3B,E, respectively). In these animals, the level of 5-HIAA increased respect to the control group in hippocampus and striatum (33% and 20%, Figure 3C,F, respectively), indicating an increase in the synthesis and metabolism of 5HT in both brain regions. The results obtained in the pineal gland of the animals receiving the experimental diet showed significant increases in 5-HTP (145% Figure 3G), 5-HT (155%, Figure 3H) and 5-HIAA (111%, Figure 3I) over controls, suggesting that foods rich in polyphenols can help restore pineal function by preventing the nocturnal decline of 5-HT (a precursor in the biosynthesis of melatonin, mediated by TPH-1) that normally occurs in the pineal gland as a result of aging [35].

Synthesis of NA was measured in hippocampus, a region rich in noradrenergic terminals; therefore, DOPA accumulated for 30 min after the inhibition of decarboxylase enzyme with NSD-1015 in hippocampus reflects the rate of NA synthesis mediated by TH. The polyphenol-rich diet increased DOPA accumulated and the NA content over controls (74% and 20%, respectively; Figure 3J,K). Moreover, the synthesis of DA mediated by TH was measured in striatum, a brain region rich in dopaminergic nerve terminals. In striatum of polyphenol fed rats, DOPA accumulated and DA content increased respect to old control rats (29% and 16%, respectively; Figure 3L,M) with a non-significant increase in the metabolite HVA (Figure 3N). 

As the levels of the metabolites analyzed reflect the recent use of the neurotransmitter, the results obtained show an increase in serotonergic neurotransmission. This statement cannot be extended to the noradrenergic system as no NA metabolite could be detected, nor dopaminergic system as the DA metabolite did not increase significantly. 

### 3.3. Effect of Polyphenol-Enriched Diet on SIRT1 Immunoreactivity in Hippocampus 

**Figure 4:**
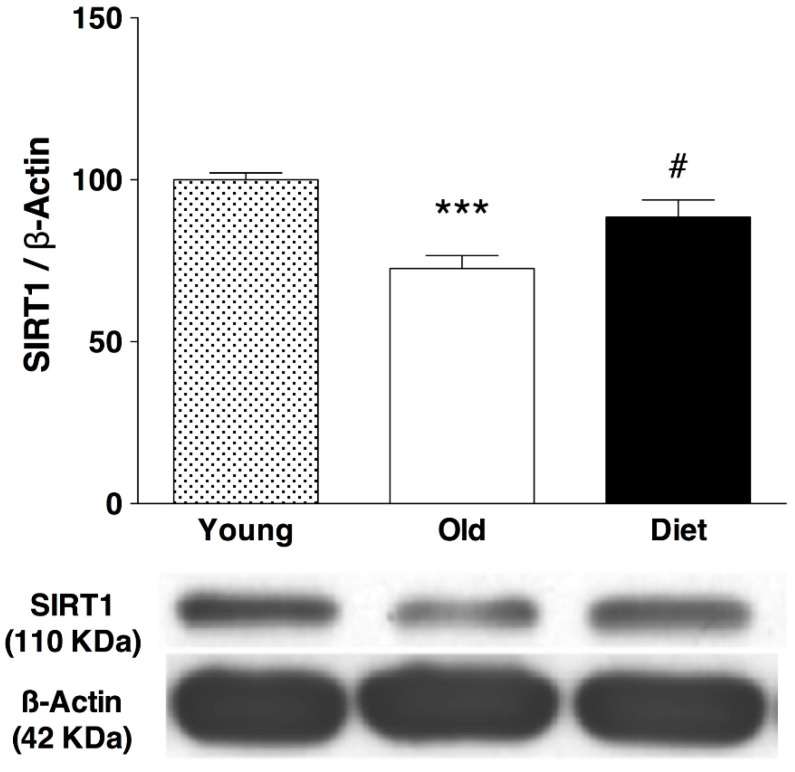
**Western blot analysis of hippocampal levels of SIRT1 after an antioxidant-enriched diet in aged rats (20 months) and comparison with young rats (3 months)**. Bars represent mean ± SEM of protein levels expressed as percentage relative to the young group. Below graph a rep-resentative immunoblot is shown. Protein level was normalized to β-actin content and each sample was analyzed in three different membranes. One-way ANOVA followed by Bonferroni post-hoc test was used for statistical analysis. One-way ANOVA detected statistical differences between the groups F(2,18) = 14.02, *p* = 0.0002; *** *p* < 0.001 when compared old control (*n* = 7) and young control animals (*n* = 7). # *p* < 0.05 when compared old animals fed with the antioxi-dant-enriched diet (*n* = 6) with old control group.

The results from the present work showed that hippocampal levels of SIRT1 protein increased at the end of treatment with a diet rich in polyphenols (22%, *p* < 0.05) over old control rats (Figure 4). A group of young rats was included in the immunoblot to test the known negative effect of age on SIRT1 levels, which has been related to the age-related decline in cognitive functions.

## 4. Discussion

The significant increase in age-related cognitive decline and chronic degenerative disorders is requiring solutions that support active and healthy aging. Here, we demonstrated the neuroprotective effect of a diet enriched in antioxidants, mainly polyphenols, improving memory and learning in older rats, accompanied by the restoration of monoaminergic neurotransmitters in the brain and the recovery of SIRT1 levels in the hippocampus.

It is now well known that normal aging of the brain is associated with neuron loss. This is reflected in decreasing concentrations of neurotransmitters and in many measures of cognition and behavior, with the hippocampus being one of the brain regions related to cognitive functions that undergoes the greatest changes with age [26]. In this way, a decline of several age-related cognitive functions in rats was associated to the decline of monoaminergic neurotransmitters and SIRT 1 protein in hippocampus [23,24]. The impairment of hippocampal function has been strongly associated with oxidative stress, induced by reduced endogenous antioxidant levels and increased reactive oxygen species (ROS) production, linked to a chronic low-grade neuroinflammatory status [4,36]. 

The results of the present work show a beneficial effect on brain function analyzed through cognitive tests in rats by a long term diet enriched in antioxidants, mainly polyphenols (also provided with anti-inflammatory properties), which have been demonstrated to regulate brain health through multiple pathways including adult neurogenesis, synaptic plasticity and neurotransmitter signaling [37]. Animals showed a better performance in 8-arms radial maze and Barnes test indicating a better visuo-spatial working memory and learning. Moreover, the results from this work demonstrated that chronic administration of polyphenol-enriched diet was enough to restore the activity of TPH-2 and TH in hippocampus and striatum, as well of TPH-1 in the pineal gland of old rats. These limiting enzymes involved in monoamine synthesis, tryptophan hydroxylase and tyrosine hydroxylase, are greatly affected by aging showing an inefficient phosphorylation caused by reactive oxygen species and cytokine injury [38,39]. This leads to a reduction in monoamine levels such as serotonin (5-HT), norepinephrine (NA) and dopamine (DA), which is believed to be partially responsible for impairments in memory and motor coordination in older [22,32,40,41,42,43], as well as the prevalence of cognitive disorders during senescence [39].

However, in the present study, the animals that consumed the antioxidant-rich diet, showed an increase in monoamine levels (5HT, NA, and DA) in regions directly involved in cognitive and motor processes such as the hippocampus and striatum [44,45,46] and also in pineal gland. These effects may be due to a protection of TPH and TH against oxidative stress as observed previously by different antioxidant molecules as melatonin [27], tocopherol [32], flavonoids [23,24] and other polyphenols [22,23]. It was also accompanied by an increase in SIRT1 protein levels in the hippocampus, which could contribute to the observed improvement in monoamines and brain functionality, since SIRT1 may interact reducing inflammation and oxidative stress. It should be noted that expression of SIRT1 decreases in the hippocampus throughout the aging process [47] as a result of high levels of oxidative stress [48]. For this reason, several studies suggest the use of antioxidants to protect the SIRT 1 protein from oxidative damage, thus preserving SIRT1 levels during aging [23,24,48,49]. This is particularly important because SIRT1 is essential for the maintenance of synaptic plasticity, memory and the attenuation of other age-related processes caused by inflammation and oxidative stress [18,48,50,51,52,53]. It is known that flavonoids act in the brain protecting vulnerable neurons and improving the function of existing neurons [54], and at least part of these beneficial effects are mediated by SIRT1. In this regard, some in vivo studies have demonstrated a SIRT1-mediated rescue of neurons by polyphenols against neurodegeneration induced by chronic excitotoxic stimuli [55] and ischemic damage [56]. These results also reinforce the relevance of the mechanism mediated by SIRT1 in the neuroprotective effect and points out the potential therapeutic role of compounds that modulate sirtuin activity in neurodegenerative disorders [51,57,58,59]. Therefore, avoiding a decrease in SIRT 1 levels or activity during the aging process, seems to have a profound impact on brain health. We have also observed an increase in the activity of the enzyme TPH-1 and consequently in the levels of 5-HT in the pineal gland of animals that consumed the polyphenol-rich diet which could be a consequence of the mechanisms mentioned above. Restoring the function of the pineal gland contributes to the maintenance of the general health of the brain. Moreover, higher intake of polyphenols affects gut microbiota profile [60], which in turn may affect brain health [61,62] in a mechanism involving attenuation of neuroinflammation [63].

In addition to the studies already mentioned, this study agrees with other works that analyze the effect of some polyphenolic compound on brain aging, revealing an overall protective effect on age-related cognitive decline. The properties of these compounds also point to a reduction in oxidative stress and/or the neuroinflammatory state as the main mechanisms [16,17,18,19,20]. Some factors related to daily life increase oxidative stress and the rate of telomere shortening, a marker of aging that has recently been associated with age-related loss of volume and functionality of the hippocampus [64]. It is now known that preventing telomere shortening can affect health and life expectancy, and that including antioxidants in the diet can contribute to this end [65]. Recently, it has been shown that a diet rich in fruits and vegetables such as the Mediterranean diet could have great potential to reduce the rate of telomere shortening in humans [66]. Along these lines, the results obtained in the present study have shown that a diet rich in antioxidants (mainly polyphenols) has preserved the functionality of the hippocampus, which could be related to the aforementioned mechanisms. Consumption of the experimental diet will increase the intake of diverse polyphenols, including flavonoids, among them anthocyanins present in red fruits. The favorable effects of these compounds on brain have been widely described [18,19,22,23,24,54]. However, it cannot be rule out that other mechanisms may have influenced the observed results, such as the possibility that the natural fruits and vegetables supplied produce an olfactory stimulation with a benefit on hippocampal circuitry of the rats [67].

Finally, this study supports that a beneficial relationship exists between a diet rich in polyphenols and brain health. Unlike other studies focusing on a single compound, the observed effects are the result of a combination of antioxidants included as a diet supplement, at least a few days a week, which enables good long-term results in terms of brain health. From our point of view, since the intake of polyphenols in humans is mainly through various fruits and vegetables, the translational value of this approach would be the possibility to include in the diet different types of fruits or other food taking into account the preferences of each person, to achieve similar results to those obtained in animals.

## Supplementary Materials

The following are available online at https://www.mdpi.com/2072-6643/13/1/59/s1, Table S1 Schedule of food, daily amounts (3 days per week) of the antioxidant-enriched diet. Figure S1 Evolution of body weight in animals fed with the experimental diet (*n* = 6) and control group (*n* = 8). Figure S2. Effects of an antioxidant-enriched diet along the chronic treatment on the rotarod test. Table S1. Schedule of food, daily amounts (3 days per week) of the antioxidant-enriched diet.

## References

[1] López-Otín C., Blasco M.A., Partridge L., Serrano M., Kroemer G. (2013). The hallmarks of aging. Cell.

[2] Harman D. (1956). Aging: A theory based on free radical and radiation chemistry. J. Gerontol..

[3] Franceschi C., Bonafè M., Valensin S., Olivieri F., De Luca M., Ottaviani E., De Benedictis G. (2000). Inflamm-aging. An evolutionary perspective on immunosenescence. Ann. N. Y. Acad. Sci..

[4] Bishop N.A., Lu T., Yankner B.A. (2010). Neural mechanisms of ageing and cognitive decline. Nature.

[5] Cruz-Sanchez F.F., Cardozo A., Castejón C., Tolosa E., Rossi M.L. (1997). Aging and the nigro-striatal pathway. J. Neural Transm..

[6] Wahl D., Cogger V.C., Solon-Biet S.M., Waern R.V.R., Gokarn R., Pulpitel T., de Cabo R., Mattson M.P., Raubenheimer D., Simpson S.J. (2016). Nutritional strategies to optimise cognitive function in the aging brain. Ageing Res. Rev..

[7] Sarris J., Logan A.C., Akbaraly T.N., Amminger G.P., Balanzá-Martínez V., Freeman M.P., Hibbeln J., Matsuoka Y., Mischoulon D., Mizoue T. (2015). Nutritional medicine as mainstream in psychiatry. Lancet Psychiatry.

[8] Kiefte-de Jong J.C., Mathers J.C., Franco O.H. (2014). Nutrition and healthy ageing: The key ingredients. Proc. Nutr. Soc..

[9] Angelino D., Godos J., Ghelfi F., Tieri M., Titta L., Lafranconi A., Marventano S., Alonzo E., Gambera A., Sciacca S. (2019). Fruit and vegetable consumption and health outcomes: An umbrella review of observational studies. Int. J. Food Sci. Nutr..

[10] Scalbert A., Johnson I., Saltmarsh M. (2005). Polyphenols: Antioxidants and beyond. Am. J. Clin. Nutr..

[11] Halliwell B., Zentella A., Gomez E., Kershenobich D. (1997). Antioxidants and human disease: A general introduction. Nutr. Rev..

[12] Khurana S., Venkataraman K., Hollingsworth A., Piche M., Tai T. (2013). Polyphenols: Benefits to the cardiovascular system in health and in aging. Nutrients.

[13] Elumalai P., Lakshmi S. (2016). Role of quercetin benefits in neurodegeneration. Adv. Neurobiol..

[14] Rahman I., Biswas S.K., Kirkham P.A. (2006). Regulation of inflammation and redox signaling by dietary polyphenols. Biochem. Pharmacol..

[15] Abbott N., Patabendige A., Dolman D., Yusof S., Begley D. (2010). Structure and function of the blood-brain barrier. Neurobiol. Dis..

[16] Liu J., Yu H., Ning X. (2006). Effect of quercetin on chronic enhancement of spatial learning and memory of mice. Sci. China C. Life Sci..

[17] Shukitt-Hale B., Cheng V., Joseph J.A. (2009). Effects of blackberries on motor and cognitive function in aged rats. Nutr. Neurosci..

[18] Spencer J. (2009). Flavonoids and brain health: Multiple effects underpinned by common mechanisms. Genes Nutr..

[19] Spencer J.P. (2010). The impact of fruit flavonoids on memory and cognition. Br. J. Nutr..

[20] Vauzour D., Vafeiadou K., Rice-Evans C., Williams R.J., Spencer J.P. (2007). Activation of pro-survival Akt and ERK1/2 signalling pathways underlie the anti-apoptotic effects of flavanones in cortical neurons. J. Neurochem..

[21] Chung S., Yao H., Caito S., Hwang J.W., Arunachalam G., Rahman I. (2010). Regulation of SIRT1 in cellular functions: Role of polyphenols. Arch. Biochem. Biophys..

[22] Sarubbo F., Ramis M.R., Aparicio S., Ruiz L., Esteban S., Miralles A., Moranta D. (2015). Improving effect of chronic resveratrol treatment on central monoamine synthesis and cognition in aged rats. Age (Dordr).

[23] Sarubbo F., Ramis M.R., Kienzer C., Aparicio S., Esteban S., Miralles A., Moranta D. (2018). Chronic Silymarin, Quercetin and Naringenintreatments increase monoamines synthesis and hippocampal Sirt1 levels improving cognition in aged tats. J. Neuroimmune Pharmacol..

[24] Ramis M.R., Sarubbo F., Tejada S., Jimenez M., Esteban S., Miralles A., Moranta D. (2020). Chronic Polyphenon-60 or Catechin treatments increase brain monoamines syntheses and hippocampal SIRT1 levels improving cognition in aged rats. Nutrients.

[25] Godos J., Caraci F., Castellano S., Currenti W., Galvano F., Ferri R., Grosso G. (2020). Association Between dietary flavonoids Intake and cognitive function in an Italian cohort. Biomolecules.

[26] Anderton B.H. (2002). Ageing of the brain. Mech. Ageing Dev..

[27] Esteban S., Garau C., Aparicio S., Moranta D., Barcelo P., Fiol M.A., Rial R. (2010). Chronic melatonin treatment and its precursor L-tryptophan improve the monoaminergic neurotransmission and related behavior in the aged rat brain. J. Pineal Res..

[28] Sharma S., Rakoczy S., Brown-Borg H. (2010). Assessment of spatial memory in mice. Life Sci..

[29] Barrett G.L., Bennie A., Trieu J., Ping S., Tsafoulis C. (2009). The chronology of age-related spatial learning impairment in two rat strains, as tested by the Barnes maze. Behav. Neurosci..

[30] Rueda-Orozco P., Soria-Gomez E., Montes-Rodriguez C., Martínez-Vargas M., Galicia O., Navarro L., Prospero-García O. (2008). A potential function of endocannabinoids in the selection of a navigation strategy by rats. Psychopharmacology.

[31] Antunes M., Biala G. (2012). The novel object recognition memory: Neurobiology, test procedure, and its modifications. Cogn. Process.

[32] Ramis M.R., Sarubbo F., Terrasa J.L., Moranta D., Aparicio S., Miralles A., Esteban S. (2016). Chronic α-tocopherol increases central monoamines synthesis and improves cognitive and motor abilities in old rats. Rejuvenation Res..

[33] Walther D., Peter J., Bashammakh S., Hörtnagl H., Voits M., Fink H., Bader M. (2003). Synthesis of serotonin by a second tryptophan hydroxylase isoform. Science.

[34] Zhang X., Beaulieu J.M., Sotnikova T.D., Gainetdinov R.R., Caron M.G. (2004). Tryptophan hydroxylase-2 controls brain serotonin synthesis. Science.

[35] Moranta D., Barcelo P., Aparicio S., Garau C., Sarubbo F., Ramis M., Nicolau C., Esteban S. (2014). Intake of melatonin increases tryptophan hydroxylase type 1 activity in aged rats: Preliminary study. Exp. Gerontol..

[36] Ryan S.M., Nolan Y.M. (2016). Neuroinflammation negatively affects adult hippocampal neurogenesis and cognition: Can exercise compensate?. Neurosci. Biobehav. Rev..

[37] Godos J., Currenti W., Angelino D., Mena P., Castellano S., Caraci F., Galvano F., Del Rio D., Ferri R., Grosso G. (2020). Diet and mental health: Review of the recent updates on molecular mechanisms. Antioxidants.

[38] De La Cruz C., Revilla E., Venero J., Ayala A., Cano J., Machado A. (1996). Oxidative inactivation of tyrosine hydroxylase in substantia nigra of aged rat. Free Radic. Biol. Med..

[39] Hussain A., Mitra A. (2000). Effect of aging on tryptophan hydroxylase in rat brain: Implications on serotonin level. Drug Metab. Dispos..

[40] Collier T., Greene J., Felten D., Stevens S., Collier K. (2004). Reduced cortical noradrenergic neurotransmission is associated with increased neophobia and impaired spatial memory in aged rats. Neurobiol. Aging.

[41] Gonzalez-Burgos I., Feria-Velasco A. (2008). Serotonin/dopamine interaction in memory formation. Prog. Brain Res..

[42] Cools R. (2011). Dopaminergic control of the striatum for high-level cognition. Curr. Opin. Neurobiol..

[43] Haider S., Saleem S., Perveen T., Tabassum S., Batool Z., Sadir S., Liaquat L., Madiha S. (2014). Age-related learning and memory deficits in rats: Role of altered brain neurotransmitters, acetylcholinesterase activity and changes in antioxidant defense system. Age (Dordr).

[44] McNeill T.H., Koek L.L., Haycock J.W. (1984). The nigrostriatal system and aging. Peptides.

[45] Burgess N., Maguire E.A., O’Keefe J. (2002). The human hippocampus and spatial and episodic memory. Neuron.

[46] Deacon R.M., Rawlins J.N. (2002). Learning impairments of hippocampal-lesioned mice in a paddling pool. Behav. Neurosci..

[47] Quintas A., de Solís A.J., Díez-Guerra F.J., Carrascosa J.M., Bogónez E. (2012). Age associated decrease of SIRT1 expression in rat hippocampus. Prevention by late onset caloric restriction. Exp. Gerontol..

[48] Wu A., Ying Z., Gómez-Pinilla F. (2006). Oxidative stress modulates Sir2α in rat hippocampus and cerebral cortex. Eur. J. Neurosci..

[49] Cao W., Dou Y., Li A. (2018). Resveratrol boosts cognitive function by targeting SIRT1. Neurochem. Res..

[50] Santangelo C., Varì R., Scazzocchio B., Di Benedetto R., Filesi C., Masella R. (2007). Polyphenols, intracellular signalling and inflammation. Ann. Ist. Super. Sanita..

[51] Gao J., Wang W.Y., Mao Y.W., Gräff J., Guan J.S., Pan L., Mak G., Kim D., Su S.C., Tsai L.H. (2010). A novel pathway regulates memory and plasticity via SIRT1 and miR-134. Nature.

[52] Salminen A., Kaarniranta K., Kauppinen A. (2013). Crosstalk between oxidative stress and SIRT1: Impact on the aging process. Int. J. Mol. Sci..

[53] Xu J., Jackson C.W., Khoury N., Escobar I., Perez-Pinzon M.A. (2018). Brain SIRT1 mediates metabolic homeostasis and neuroprotection. Front. Endocrinol. (Lausanne).

[54] Spencer J.P.E. (2008). Food for thought: The role of dietary flavonoids in enhancing human memory, learning and neuro-cognitive performance. Proc Nutr Soc..

[55] Lazo-Gomez R., Tapia R. (2017). Quercetin prevents spinal motor neuron degeneration induced by chronic excitotoxic stimulus by a sirtuin 1-dependent mechanism. Transl. Neurodegener..

[56] Della-Morte D., Dave K.R., DeFazio R.A., Bao Y.C., Raval A.P., Perez-Pinzon M.A. (2009). Resveratrol pretreatment protects rat brain from cerebral ischemic damage via a sirtuin 1-uncoupling protein 2 pathway. Neuroscience.

[57] Qin W., Yang T., Ho L., Zhao Z., Wang J., Chen L., Pasinetti G. (2006). Neuronal SIRT1 activation as a novel mechanism underlying the prevention of Alzheimer disease amyloid neuropathology by calorie restriction. J. Biol. Chem..

[58] Jiang M., Wang J., Fu J., Du L., Jeong H., West T., Duan W. (2012). Neuroprotective role of Sirt1 in mammalian models of Huntington’s disease through activation of multiple Sirt1 targets. Nat. Med..

[59] Herskovits A., Guarente L. (2013). Sirtuin deacetylases in neurodegenerative diseases of aging. Cell Res..

[60] Carrera-Quintanar L., López Roa R.I., Quintero-Fabián S., Sánchez-Sánchez M.A., Vizmanos B., Ortuño-Sahagún D. (2018). Phytochemicals that influence gut microbiota as prophylactics and for the treatment of obesity and inflammatory diseases. Mediators Inflamm..

[61] Ceppa F., Mancini A., Tuohy K. (2019). Current evidence linking diet to gut microbiota and brain development and function. Int. J. Food Sci. Nutr..

[62] Salvucci E. (2019). The human-microbiome superorganism and its modulation to restore health. Int. J. Food Sci. Nutr..

[63] Carregosa D., Carecho R., Figueira I., Santos C.N. (2020). Low-molecular weight metabolites from polyphenols as effectors for attenuating neuroinflammation. J. Agric. Food Chem..

[64] Palmos A.B., Duarte R.R.R., Smeeth D.M., Hedges E.C., Nixon D.F., Thuret S., Powell T.R. (2020). Telomere length and human hippocampal neurogenesis. Neuropsychopharmacology.

[65] Galiè S., Canudas S., Muralidharan J., García-Gavilán J., Bulló M., Salas-Salvado J. (2020). Impact of nutrition on telomere health: Systematic review of observational cohort studies and randomized clinical trials. Adv. Nutr..

[66] Canudas S., Becerra-Tomás N., Hernández-Alonso P., Galié S., Leung C., Crous-Bou M., De Vivo I., Gao Y., Gu Y., Meinilä J. (2020). Mediterranean diet and telomere length: A systematic review and meta-analysis. Adv. Nutr..

[67] Rusznák Z., Sengul G., Paxinos G., Kim W.S., Fu Y. (2018). Odor enrichment increases hippocampal neuron numbers in mouse. Exp. Neurobiol..

